# Hollow Spherical Capsules From Geopolymerized Gel Beads With Halloysite Nanotubes for Pollutants Removal and CO_2_ Capture

**DOI:** 10.1002/smll.202504306

**Published:** 2025-06-17

**Authors:** Alessandro Lo Bianco, Martina Maria Calvino, Giuseppe Cavallaro, Pavel Šiler, Jaromìr Wasserbauer, Stefana Milioto, Giuseppe Lazzara

**Affiliations:** ^1^ Department of Physics and Chemistry “Emilio Segrè” University of Palermo Viale delle Scienze 17 Palermo 90128 Italy; ^2^ Faculty of Chemistry, Institute of Materials Science Brno University of Technology Purkyňova 118 Brno 61200 Czech Republic

**Keywords:** alginate, composite gel beads, geopolymer, halloysite nanotubes, remediation

## Abstract

A scalable protocol is proposed for the fabrication of hollow spherical capsules by geopolymerization of halloysite clay nanotubes (HNTs) incorporated within alginate gel beads. Alginate/HNTs composite beads have been geopolymerized by alkaline treatment through their immersion in highly concentrated NaOH solution. The influence of the immersion time on the structure and mesoscopic properties of the beads has been studied to optimize the geopolymerization efficacy as well as the mechanical resistance and adsorption performances of the capsules. SEM images demonstrate that the alkaline treatment is efficient in the conversion of compact beads to hollow spherical capsules, which possess improved water uptake capacity and enhance flexibility under compressive forces as evidenced by the relevant increase (in the range ca. 55–75%) of the elastic modulus. Due to their internal cavity and surface porosities, geopolymerized capsules exhibit better adsorption performances toward hydrocarbons with respect to the untreated alginate/HNTs beads. Moreover, geopolymerization of alginate/HNTs beads significantly improves the CO_2_ capture efficiency. The CO_2_ amount adsorbed by the composites bead has been doubled after 5 seconds of alkaline treatment. This study highlights that the geopolymerization of halloysite loaded in biopolymeric beads can be exploited to obtain sustainable materials suitable for CO_2_ storage and removal of contaminants.

## Graphical abstract

Hollow spherical microparticles have been prepared by geopolymerization of alginate/halloysite clay beads. The microspheres are efficient for CO_2_ capture and removal of hydrocarbons pollutants.

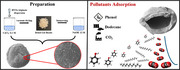

## 1 Introduction

The growing demand for materials capable of adsorbing environmental pollutants has led researchers to develop innovative composites with improved adsorption capacities. Aliphatic and aromatic hydrocarbons are critical pollutants due to their persistence and toxicity. On the other hand, CO_2_ drives climate change because of the greenhouse effect,^[^ ^1^, ^2^ ^]^ prompting global efforts like the Paris Agreement to limit temperature increases below 2 °C.^[^ ^3^ ^]^ Effective materials for CO_2_ capture are essential to mitigate its impact.^[^ ^4^, ^5^, ^6^, ^7^, ^8^ ^]^ Hydrocarbons, such as dodecane and phenolic compounds, contaminate air and water and pose serious risks to health and ecosystems.^[^ ^9^, ^10^, ^11^ ^]^ Regulatory agencies like the EU and EPA classify them as priority pollutants, requiring strict removal standards, including limiting phenol levels in drinking water to below 0.1 µg L^−1^,^[^ ^12^, ^13^ ^]^ to protect aquatic life and human health. Developing materials that can selectively and efficiently adsorb these compounds is crucial for mitigating their impact and restoring environmental balance. Geopolymers synthesized by reacting aluminosilicate sources with alkaline solutions have demonstrated significant potential as environmentally friendly^[^ ^14^, ^15^, ^16^, ^17^ ^]^ and efficient adsorbents.^[^ ^18^, ^19^ ^]^ These inorganic polymers are characterized by their high thermal stability,^[^ ^20^, ^21^ ^]^ chemical resistance,^[^ ^22^ ^]^ and porosity^[^ ^23^ ^]^ making them versatile for applications ranging from water treatment to CO_2_ capture.^[^ ^24^ ^]^ However, most current geopolymer studies focus on large‐scale applications like construction, and only a few investigate the potential of geopolymers in pollutant adsorption field. One promising approach involves integrating halloysite clay nanotubes (HNTs) into composite materials as precursors for geopolymers. Halloysite is a naturally occurring clay mineral with a unique tubular structure.^[^ ^25^, ^26^, ^27^, ^28^ ^]^ It usually shows external diameters between 20 and 200 nm, inner diameters ranging from 10 to 50 nm, and an average length of approximately 1 µm.^[^ ^29^ ^]^ This tubular structure provides distinct adsorption sites, with a positively charged inner cavity and a negatively charged outer surface, which enables selective loading and controlled release of active substances,^[^ ^30^, ^31^, ^32^, ^33^, ^34^, ^35^ ^]^ catalysis^[^ ^36^, ^37^, ^38^, ^39^, ^40^, ^41^ ^]^ and pollutant adsorption.^[^ ^42^, ^43^, ^44^, ^45^ ^]^ Gel beads, formed by cross‐linking polymers like alginate, are versatile materials extensively used in environmental applications. Their robust matrix with tunable properties enables diverse functionalities, such as sequestrant and drug delivery capabilities.^[^ ^46^, ^47^, ^48^ ^]^ Alginate, a biodegradable and biocompatible biopolymer derived from brown seaweed,^[^ ^49^, ^50^ ^]^ is particularly valued for its ability to form hydrogels through interactions with divalent cations.^[^ ^51^, ^52^, ^53^ ^]^

In this work, we investigated the geopolymerization process of alginate/halloysite composite beads to fabricate hollow spherical particles suitable as adsorbent materials. Literature reports the formation of hollow structures based on geopolymer composites, such as glazed hollow beads filled with polypropylene fibres,^[^ ^54^ ^]^ fly‐ash/slag‐based hollow glass microspheres.^[^ ^55^ ^]^ and fly ash/phosphate geopolymer hollow spheres.^[^ ^56^ ^]^ These materials were obtained by immersion in NaOH solution for a prolonged time, such as 24 hours, which is typical for geopolymerization processes as reported in a recent review.^[^ ^57^ ^]^ We recently explored shorter alkaline activation treatments (10 seconds) for the geopolymerization of inorganic films based on Patch halloysite nanotubes.^[^ ^24^ ^]^ Here, we investigated for the first time the formation of hollow geopolymeric structures by exploiting a short alkaline treatment (5 seconds) of alginate/halloysite bead. It should be noted that the geopolymerization of biopolymer/halloysite gel beads was not studied. By integrating halloysite nanotubes into the alginate matrix and applying two different times of geopolymerization, this study investigates how these processes influence the surface characteristics and internal structure of composite beads. The findings aim to advance the design of optimized adsorbents for environmental remediation, industrial separations, and greenhouse gas mitigation, demonstrating the potential of geopolymer‐alginate gel bead systems as customizable and effective solutions for targeted pollutant removal.

## 2 Experimental Section

### 2.1 Materials

Alginic acid sodium salt (Mw = 70‐100 kDa), and sodium hydroxide are Sigma Aldrich products. Halloysite nanotubes (Al_2_Si_2_O_5_(OH)_4_·2H_2_O), also supplied by Sigma‐Aldrich, were sourced from Dragon Mine geological deposit in Utah, USA. Calcium chloride dihydrate (CaCl_2_·2H_2_O) was a Merck product. Dodecane and phenol are Fluka Chemica products.

### 2.2 Preparation of Alginate/HNTs Composite Beads

**Figure 1:**
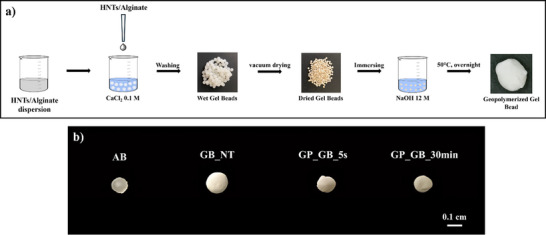
a) Illustration GP_ of the Gel Beads preparation protocol and b) optical images of AB, GB_NT, GB_5s GP_GB_5s and GP_GB_30 min samples.

Gel Beads were prepared using the dropping technique as described elsewhere.^[^ ^58^ ^]^ First, a 2 wt% sodium alginate dispersion in water was prepared by dissolving the polymer through magnetically stirring for 2 hours at room temperature. Subsequently, a proper amount of halloysite nanotubes was added to the polymer dispersion allowing to obtain an alginate/HNTs mixture with a 1:1 mass ratio. The stabilization of the aqueous mixture was achieved by stirring for 2 hours at 25 °C. The alginate/HNTs dispersion was dispensed into a 0.1 M CaCl_2_ solution using a peristaltic pump equipped with a 0.4 mm diameter nozzle. The distance between the nozzle and CaCl_2_ solution was fixed at 5 cm. The alginate/HNTs gel beads (GB_NT) were kept in 0.1 M CaCl_2_ solution for 24 hours to ensure the complete cross‐linking of the biopolymer. Then, the composite beads were subjected to three cycles of washing in distilled water to remove any excess chloride ions and subsequently dried under vacuum at 25 °C. For comparison, gel beads based on neat alginate (AB) were prepared using the same procedure. **Figure** **1** displays a schematic illustration of the preparation protocol as well as macroscopic images of AB and GB_NT beads.

### 2.3 Geopolymerization of Alginate/HNTs Composite Beads

As displayed in Figure 1, the geopolymerization of dried GB_NT beads was conducted by their immersion within NaOH 12 M solution. The effect of the immersion time on the geopolymerization process was investigated. In detail, the composite beads were kept immersed in NaOH 12 M solution for 5 seconds and 30 minutes. The corresponding geopolymerized beads are identified as GP_GB_5s and GP_GB_30 min, respectively. Afterward, the beads were cured at 50 °C for 24 hours and washed with water by several cycles to remove any excess of sodium hydroxide. The complete removal of NaOH was confirmed by monitoring the pH of the rinsed water. Finally, the geopolymerized beads were dried overnight at 50 °C.

### 2.4 Methods

#### 2.4.1 Thermogravimetric Analysis (TGA)

Thermogravimetric experiments were carried out by a TGA550 apparatus (TA Instruments) under inert atmosphere using 60 and 40 cm^3^ min^−1^ Nitrogen flows for the sample and the balance, respectively. Samples were heated in a platinum pan from room temperature to 800 °C with a scanning rate of 20 °C min^−1^.

#### 2.4.2 Fourier Transform Infrared (FTIR) Spectroscopy

FT‐IR spectra were collected from 4000 to 400 cm⁻¹ using a Perkin‐Elmer FT‐IR Spectrum One instrument. Experiments were conducted at room temperature with a spectral resolution of 4 cm⁻¹ on KBr pellets with a low concentration (<2wt%) of the milled beads.

#### 2.4.3 Scanning Electron Microscopy (SEM)

Morphological investigations were conducted by using a Scanning Electron microscope (Zeiss EVO LS 10). Secondary electron mode with an acceleration voltage of 15 kV and a working distance of 12 mm were used. All samples were sputtered with gold to avoid surface charging and improve the image quality.

#### 2.4.4 Dynamic Mechanical Analysis (DMA)

Dynamic mechanical analysis was conducted with a DMA Q800 (TA Instruments) using a clamp for compression with a cylindrical geometry. Compressive tests were performed in stress ramp mode at 25 °C. The stress ramp was set at 1 MPa min^−1^ up to breaking point. The experiments were repeated five times, and the average values with the corresponding relative errors are reported.

#### 2.4.5 Water Uptake Measurements

Water uptake measurements were conducted by placing the gel bead samples inside a desiccator with a 99% of relative humidity. The water uptake (WU%) values at different equilibration times were calculated by monitoring the mass of the beads as follows

(1) WU%=100·((mt−w−mi)/mi)

where m_i_ was the initial mass, while m_t‐w_ was the mass after a certain equilibration time at 99% of relative humidity.

The measurements were repeated five times, and the average values with the corresponding relative errors were presented.

#### 2.4.6 CO_2_ Capture Tests

The CO_2_ capture capacity of the beads was investigated by thermogravimetry using a TGA550 Discovery Series (TA Instruments) apparatus. Initially, the beads were equilibrated at 100 °C for 15 minutes under Nitrogen atmosphere to remove any moisture. Then, the temperature was shifted to 35 °C for 10 minutes keeping inert conditions. Finally, the gas was switched to CO_2_ under isothermal conditions (T = 35 °C). We monitored the mass change during the last step for 200minutes. Flow rates for both Nitrogen and CO_2_ gases were set at 60 and 40 cm^3^ min^−1^ for the sample and the balance, respectively.

#### 2.4.7 Absorption of Dodecane from Vapor Phase

The absorption efficiency of the beads towards dodecane from vapour phase was gravimetrically determined. To this purpose, the gel beads were kept in a desiccator saturated with dodecane vapours for variable times. The dodecane uptake (DU%) of the beads was calculated using the following equation

(2) DU%=100·((mt−d−mi)/mi)

being m_i_ the initial mass, while m_t‐d_ was the mass after a certain equilibration time within the desiccator saturated by dodecane vapours.

The measurements were repeated five times, and the average values with the corresponding relative errors were reported.

Cyclic experiments were carried out after 48 hours of dodecane vapours absorption onto GP_GB_5s. The regeneration of GP_GB_5s was conducted by keeping the geopolymerized bead under vacuum conditions for 12 hours, which assures the complete desorption of dodecane.

#### 2.4.8 Removal of Phenol from Aqueous Phase

The removal capacity of the beads towards phenol dissolved in aqueous solvent was studied by UV–vis spectrophotometry using Analytikjena Specord S600. In particular, we placed a bead within the quartz cuvettes containing 2 mL of phenol solution in water. The phenol concentration was set at 0.0255 g L^−1^. Then, we collected the spectra from 200 to 700 nm as a function of the time. The phenol removal efficiency was estimated from the absorbance of the peak centred at 270 nm. The experiments were conducted were conducted at a controlled temperature of 25.0 ± 0.1 °C.

Cyclic experiments were carried out after 25 hours of phenol removal from GP_GB_5s. The regeneration of GP_GB_5s was conducted by keeping the geopolymerized bead in water for 25 hours, which guarantees the complete desorption of phenol.

## 3 Results and Discussion

### 3.1 Geopolymerization of Alginate/HNTs Beads: TGA and FTIR Spectroscopy

The geopolymerization of alginate/HNTs gel beads by their immersion in NaOH solution was demonstrated by both thermogravimetry and FTIR spectroscopy.

**Figure 2:**
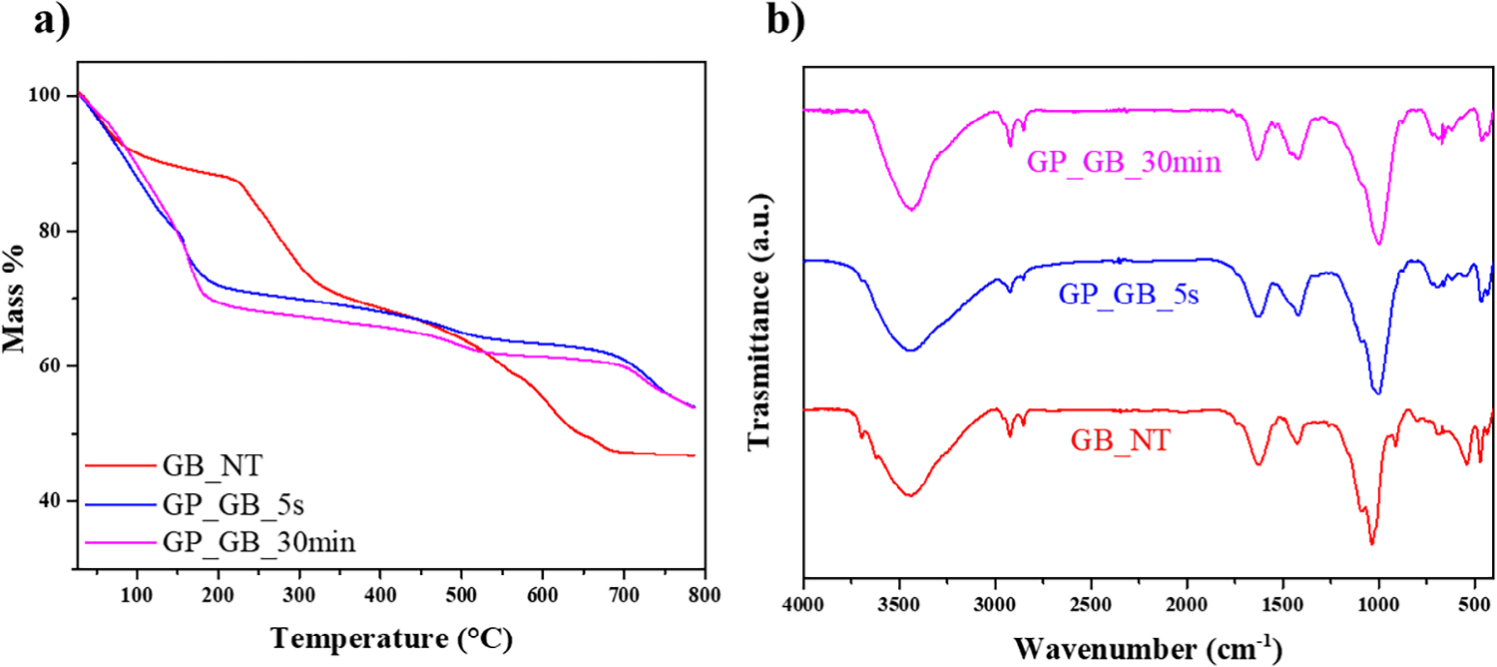
a) Thermogravimetric curves of GB_NT, GP_GB_5s and GP_GB_30 min. b) FT‐IR spectra of GB_NT, GP_GB_5s and GP_GB_30 min.

**Figure** **2**a shows thermogravimetric (TG) curves of gel beads filled with halloysite before and after their activation by NaOH.

As a general result, TG curves evidenced a mass loss from 25 to 150 °C that can be attributed to the water molecules physically adsorbed on the gel beads. Similar considerations are reported for biopolymer/clay composite films before and after their geopolymerization.^[^ ^59^ ^]^ We detected that the treatment of GB_NT within NaOH solution for both immersion times (5 seconds and 30 minutes) induces a relevant increase of the moisture loss. Similar results were detected after geopolymerization of halloysite cellulose/HNT composite^[^ ^59^ ^]^ and inorganic film based on patch halloysite.^[^ ^24^ ^]^ As shown in Figure 2a, GB_NT exhibited a clear mass reduction in the range between 450 and 700 °C corresponding to the dehydroxylation of the aluminum inner sheets of halloysite.^[^ ^59^ ^]^ Oppositely, this signal was not detected for both GP_GB_5s and GP_GB_30 min samples confirming the successful geopolymerization.^[^ ^24^, ^60^ ^]^ Moreover, GB_NT showed a mass loss at 200–350 °C that can be attributed to alginate decomposition due to breakdown of glycosidic bonds as well as decarboxylation and decarbonylation of biopolymer.^[^ ^61^ ^]^ Accordingly, the gel bead based on pristine alginate exhibited a relevant mass change within the same temperature interval (see Supporting Information). As compared with GB_NT, the alginate degradation process was shifted to a lower temperature region (150–200 °C) in the geopolymerized gel beads indicating that halloysite activation by NaOH may destabilize the biopolymer structure. Namely, the conversion of halloysite nanotubes to geopolymers can accelerate the thermal decomposition of alginate. **Table** **1** collects the alginate degradation temperature (estimated from the onset point of TG curves) for all gel beads.

**Table 1:** Onset temperature for alginate degradation and mass loss calculated from room temperature to 150 °C (ML_150_) for gel bead samples.

Sample	ML_150_ [%]	T_onset_ [°C]
AB	11.6 ± 0.2	199 ± 3
GB_NT	10.4 ± 0.2	226 ± 4
GP_GB_5s	20.3 ± 0.4	156 ± 3
GB_GB_30min	20.4 ± 0.4	160 ± 3

The effects of geopolymerization on the chemical structures of alginate/HNTs beads were evaluated by FT‐IR spectra (Figure 2b). We detected that GB_NT possesses FT‐IR peaks at 3697 cm^−1^ and 3623 cm^−1^ that correspond to hydroxyl group vibrations of the inner and outer surfaces of halloysite nanotubes in agreement with literature.^[^ ^62^ ^]^ Accordingly to the successful geopolymerization, the characteristic FT‐IR peaks of halloysite at ca. 3600 cm^−1^ were not observed in GP_GB_5s and GP_GB_30 min samples.^[^ ^63^ ^]^ The geopolymerization was demonstrated also by the analysis of the signal due to the asymmetric Si−O−T stretching (T being either Si or Al in tetrahedral form), which is centred at 1044 cm^−1^ in the GB_NT material. This band was shifted to 1012 cm^−1^ in both GP_GB_5s and GP_GB_30 min due to the structural reorganization of halloysite and the larger aluminium content in the silicate framework of geopolymers.^[^ ^64^, ^65^, ^66^ ^]^

A complete description of the main FT‐IR signals for all samples is reported in Supporting Information (Table S1, Supporting Information).

In conclusion, we can state that both TGA and FT‐IR spectroscopy evidenced that the NaOH treatment was effective to achieve the geopolymerization of alginate/HNT beads. Moreover, both techniques highlighted that geopolymerized materials can be obtained using a short immersion time (5 seconds).

### 3.2 Effects of Geopolymerization on the Morphology of Alginate/HNTs Composite Beads

**Figure 3:**
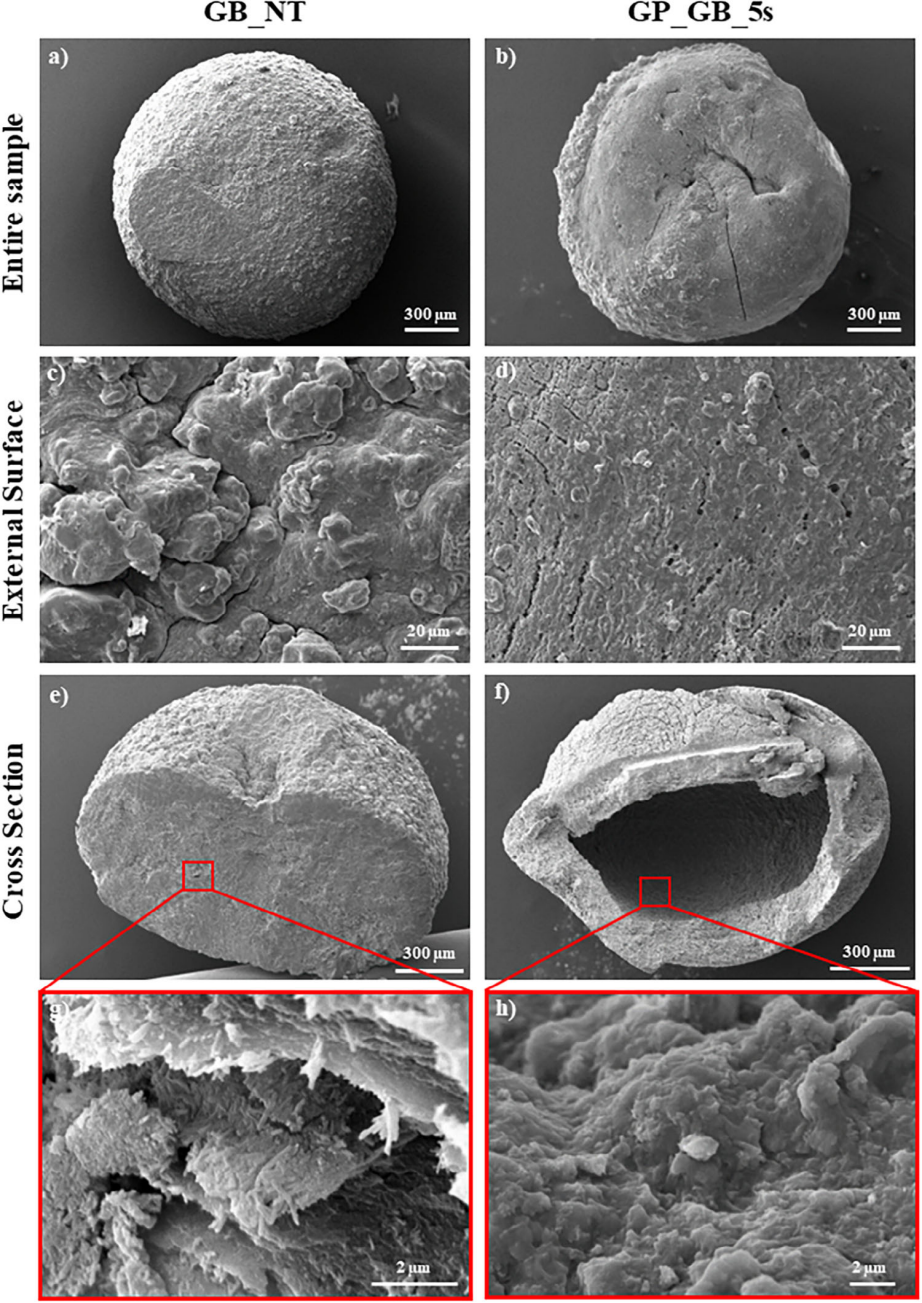
SEM images of GB_NT a,c,e,g) and GP_GB_5s b,d,f,h). The entire sample (a,b), the external surface (c,d), the cross‐section e–h).

**Figure** **3** compares SEM images of alginate/HNT beads before and after their alkaline treatment for 5 seconds.

As shown by SEM images of the entire beads (Figure 3a,b), the untreated GB_NT sample showed a more regular shape and higher roughness with respect to those of GP_GB_5s. The micrographs at higher magnification (Figure 3c,d) evidenced that the external surface of GB_NT bead is compact with the presence of irregular clusters randomly distributed, while GP_GB_5s possesses a porous structure with an average pore diameter of 1.4 µm. Interestingly, the GB_NT sample did not show any nanotubes exposed on the surface (Figure 3c) suggesting that halloysite clay penetrates within the bead structure due to their good affinity with alginate matrix. SEM images of the cross section (Figure 3e–h) revealed that the geopolymerization process changes the internal structure of alginate/HNT gel bead. Contrary to the untreated gel bead, the GP_GB_5s sample possesses a cavity of about 950 µm with an irregular and rough surface resulting in a coarse and uneven texture (Figure 3f). The internal surface of the geopolymerized bead is composed by a mixture of alginate matrix and geopolymeric material. The formation of the cavity in the geopolymerized gel bead might be induced by the following factors: 1) the alkaline activation of halloysite induces the shrinkage of the clay nanotubes due to loss of water and reorganization of the structure.^[^ ^68^, ^69^ ^]^ Therefore, the total specific pore volume of halloysite is reduced by geopolymerization because the hollow tubular structure of the clay is lost. Being that halloysite nanotubes are mostly distributed within the core of GB_NT, we can expect that their conversion to geopolymers can generate an empty space within the core of the bead generating an internal cavity; 2) the crosslinking degree of alginate is lower in the core of GB_NT with respect to the external surface in agreement with literature.^[^ ^70^, ^71^ ^]^ Consequently, the internal part of the alginate/HNT bead is more sensitive to structural changes; 3) the removal of the excess of sodium hydroxide by washing cycles after the GB_NT immersion may contribute to the cavity formation in the GP_GB_5s sample. The complete removal of NaOH from the geopolymerized gel bead was achieved after multiple washing cycles evidencing that the NaOH release from GP_GB_5s is slow. Accordingly, we detected a gradual decrease of the pH of the rinsed water (from 11 to 7) during consecutive washing cycles of the geopolymerized beads. The pH variations of rinsed water during six washing cycles of GP_GB_5s sample are reported in Supporting Information (Figure S1, Supporting Information). This could indicate that sodium hydroxide is mainly confined in the core of the composite bead. The combination of these factors can contribute to the formation of the internal cavity in the geopolymerized alginate/halloysite beads. Literature reports that geopolymer/alginate beads with honeycomb‐like cavities can be produced using a different method, where a geopolymer slurry was directly mixed with an alginate solution and then crosslinked through a dropping method in a CaCl_2_ solution.^[^ ^72^ ^]^ It should be noted that gel beads composed by pristine alginate possess different morphological characteristics with respect to both HNTs addition and the subsequent alkaline activation generated gel beads with different morphologies with respect to both GB_NT and GP_GB_5s samples. As shown by SEM images (see Figure S2 in Supporting Information), the cross‐section of AB samples is significantly smoother and more uniform as compared to the composite materials highlighting the relevant impact of the halloysite addition and consequent geopolymerization on the internal structure of alginate‐based beads

### 3.3 Influence of Geopolymerization on the Compressive Properties and Water Uptake of the Alginate/HNTs Beads

**Figure 4:**
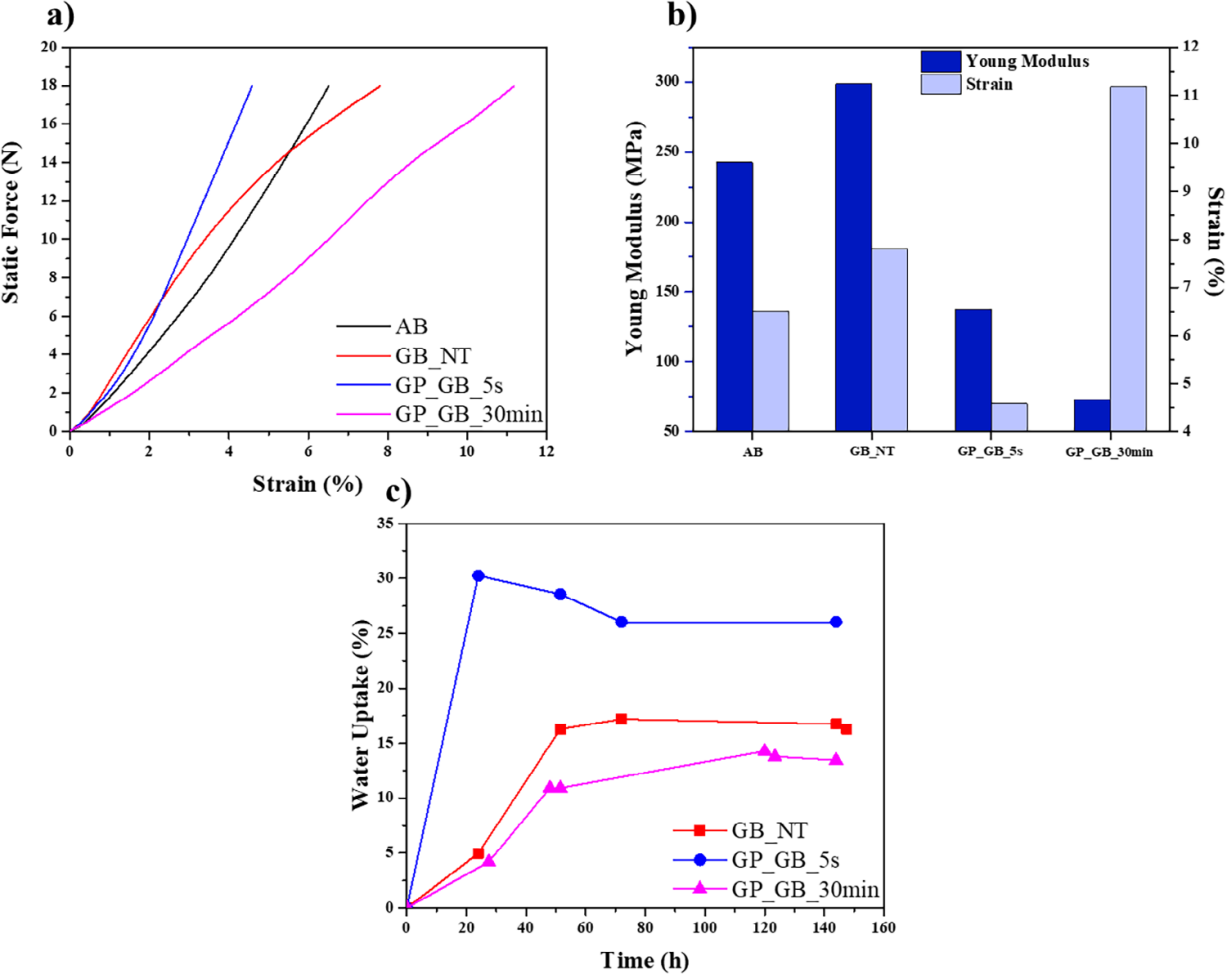
Mechanical properties of Gel beads samples. a) Static Force versus strain curve obtained by compression tests. b) Young's modulus and strain data at maximum applied force (18 N). The relative error for both Young modulus and strain data is 5%. c) Water uptake as a function of time for GB_NT, GP_GB_5s, and GP_GB_30 min samples. The relative error for water uptake is < 0.5%.

To evaluate the suitability of geopolymerized beads in technological applications, we investigated that influence of the alkaline treatment on the compressive properties and water uptake of alginate/HNTs sample. **Figure** **4**a shows the static force versus strain curves of all the prepared beads obtained by compression tests performed through DMA. It should be noted that the compression experiments were performed up to 18 N, which is the maximum force allowed by DMA Q800 (TA Instruments).

As a general result, we detected that all alginate‐based beads do not break during DMA experiments. Based on the quantitative analysis of DMA curves, we determined the Young modulus and the ultimate percent strain of the investigated beads (Figure 4a). The Young modulus of GB_NT is larger with respect to that of AB sample indicating that the addition of halloysite nanotubes within alginate matrix caused an increase of the rigidity of the biopolymeric bead. This effect is consistent with the compact structure evidenced by SEM images of GB_NT sample (Figure 3). The subsequent geopolymerization of GB‐NT sample induced significant reductions of the Young modulus highlighting that the geopolymerized beads are more elastic and flexible. In particular, we calculated that the Young modulus was decreased by 54.1% and 75.6% for alkaline treatments of 5 seconds and 30 minutes, respectively. The enhancement of the flexibility could be related to the morphological characteristics of the geopolymerized beads, which present an internal cavity and a porous structure (Figure 3). Regarding the ultimate percent strain percentage, we detected that pure alginate sample (AB) exhibits less deformation under compression as compared to GB_NT. This suggests that while the addition of halloysite increases stiffness, it also allows for greater deformation under stress compared to the pure alginate beads. The differences in mechanical properties across the samples can be largely attributed to their diverse internal morphologies. GP_GB_5s benefits from its internal cavity and porosity, which not only makes it more flexible but also enhances its resistance to compressive stress.^[^ ^73^, ^74^ ^]^ On the other hand, the compactness of the halloysite nanotubes in GB_NT increases the overall rigidity of the material resulting in reduced elasticity. The GP_GB_30 min sample, while demonstrating greater elastic behaviour compared to the other samples, also exhibits the highest strain value. This response may be linked to the extended duration of the alkaline treatment, which could degrade the outer alginate structure,^[^ ^75^ ^]^ thereby influencing the sample's mechanical response. In addition, we calculated the stored energy values at 18 N (see in Table S2, Supporting Information) by integration of static force versus strain curves. Interestingly, GP_GB_30 min exhibited the highest energy storage capacity, with values approximately 1.7 and 2.5 times greater than those of GB_NT and AB, respectively. On the other hand, GP_GB_5s showed the lowest stored energy among all samples.

Figure 4c shows the water uptake of the beads over time, and it clearly demonstrates that the GP_GB_5s sample exhibits the highest water absorption capacity. This sample reached its maximum water uptake (ca. 26%) within 24 hours. On the other hand, we calculated that the water uptakes at saturation are ca. 17% and ca. 13% for GB_NT and GP_GB_30 min, respectively. The differences in water absorption capacity between the beads can be attributed to the structural changes induced by the geopolymerization process. The 5‐second geopolymerization treatment in GP_GB_5s notably enhanced the water adsorption capacity, likely due to the development of a highly porous structure and the formation of an internal cavity. This morphology significantly increased the surface area available for interaction with water molecules. Additionally, the intrinsic hydrophilicity of geopolymer materials^[^ ^76^ ^]^ and alginate with his hydroxyl groups^[^ ^77^, ^78^ ^]^ further promotes efficient water uptake, making this treatment particularly effective in optimizing absorption performance. In contrast, the GP_GB_30 min sample, despite undergoing geopolymerization, exhibits a lower water absorption capacity. This reduction may result from degradation of the alginate chains caused by prolonged exposure to the highly alkaline environment during the 30‐minute treatment, which could compromise its structural integrity limiting its ability to retain water.^[^ ^75^ ^]^ Similarly, the untreated GB_NT sample, while retaining a moderate water uptake due to halloysite hydrophilicity, lacks the enhanced porosity and internal cavities seen in the geopolymerized sample, resulting in lower absorption efficiency compared to GP_GB_5s. Overall, the structural modifications induced by the 5‐second geopolymerization treatment in GP_GB_5s provide a clear advantage in terms of water uptake performance.

### 3.4 Absorption Capacity of Geopolymerized Beads towards Hydrocarbons

We tested the absorption capacity of the alginate/HNTs beads (before and after geopolymerization) towards hydrocarbons to evaluate their suitability as removal agents of pollutants in both vapour and aqueous phases. In particular, we studied the removal efficiencies of the beads toward dodecane (aliphatic hydrocarbon) vapours and phenol (aromatic hydrocarbon) dissolved in water.

#### 3.4.1 Absorption Efficiency toward Dodecane Vapors

**Figure 5:**
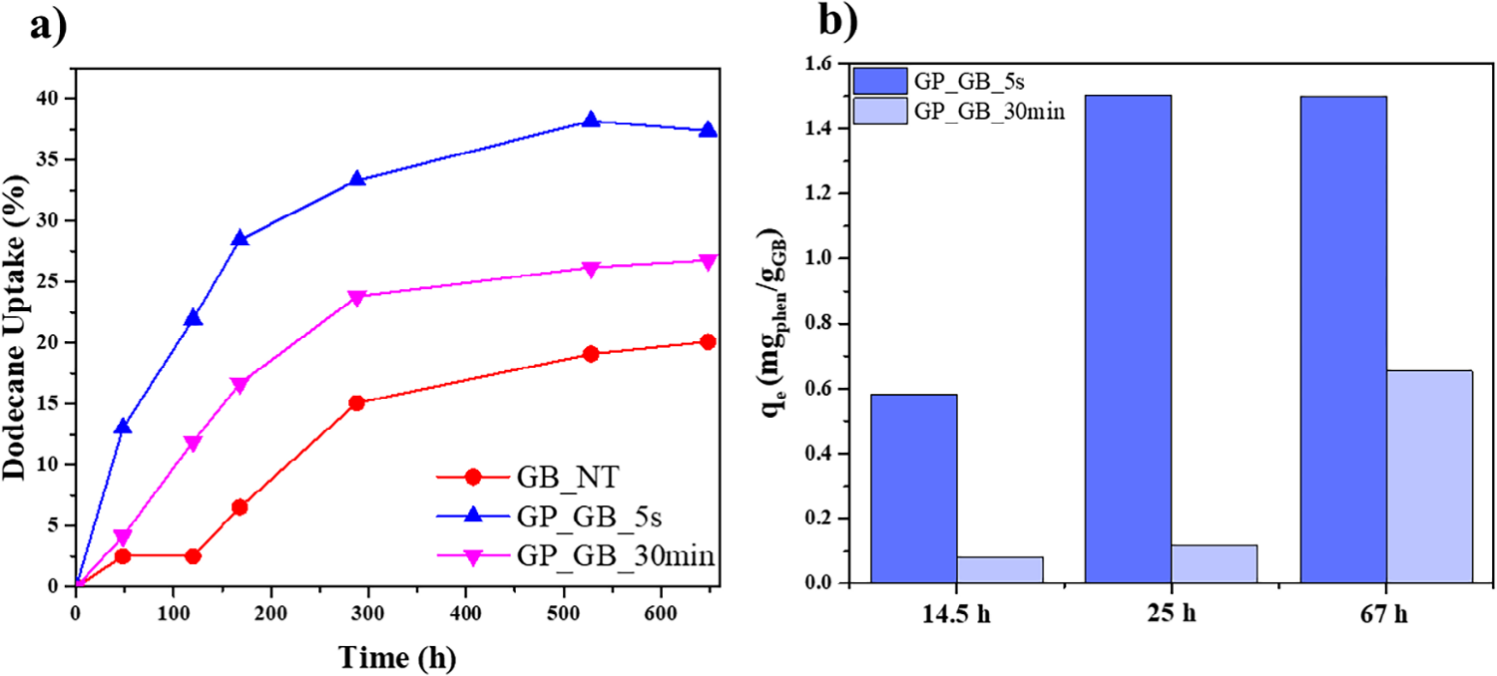
a) Dodecane uptake as a function of time for GB_NT, GP_GB_5s, and GP_GB_30 min samples. b) q_e_ (mg_phen_/g_GB_) values of GP_GB_5s and GP_GB_30 min in three different times of adsorption of Phenol in solution. The relative error for both dodecane uptake and q_e_ values was found to be < 0.5%.

**Figure** **5**a displays the dodecane uptake over time for the gel bead samples, revealing a significant difference in absorption capacities between the geopolymerized samples and the untreated GB_NT sample. Notably, the geopolymerized beads showed a much higher capacity to absorb dodecane. After 48 hours, the uptake values are 13.01% for the GP_GB_5s sample, 4.17% for the GP_GB_30 min sample, and 2.51% for the untreated GB_NT sample. From the initial stages of analysis, GP_GB_5s showed also faster absorption kinetics compared to the other samples suggesting an enhanced affinity and interaction with dodecane. The absorption trend for all the samples is initially rapid, showing a significant increase up to 288 hours with dodecane uptake levels of 33.3%, 23.8%, and 15.1% for GP_GB_5s, GP_GB_30 min, and GB_NT, respectively. These values slowly increased until the saturation conditions were reached. The maximum uptake values observed at equilibrium are 38.2% for GP_GB_5s, 26.8% for GP_GB_30 min, and 20.1% for GB_NT. These results indicated that the geopolymerized samples, particularly GP_GB_5s, exhibit enhanced dodecane uptake capacities with respect to GB_NT. This can be attributed to their increased porosity and larger surface area, likely due to the internal cavity structure developed during the geopolymerization process. The porous structure facilitates the interaction with dodecane molecules, allowing for more efficient adsorption. Additionally, the rapid uptake kinetics in the GP_GB_5s sample further supports the role of microstructural modifications in promoting faster and more extensive hydrocarbon adsorption as reported for the adsorption of gaseous pollutants,^[^ ^79^, ^80^ ^]^ further showcasing their versatility in environmental remediation. This suggests that optimizing the geopolymerization protocol could allow for the tailored design of materials with specific uptake properties for various hydrocarbons.

As concern GP_GB_5s, we performed a cyclic experiment after 48 hours of dodecane absorption to evaluate the potential of the geopolymerized bead for practical applications. We estimated that the absorption efficiency is slightly reduced (12.2%) after the regeneration of geopolymerized gel bead in vacuum conditions. This result is promising for the use of GP_GB_5s as recycling adsorbent material of hydrocarbon vapours.

#### 3.4.2 Removal of Phenol from Aqueous Phase

The removal efficiency of the gel beads towards phenol dissolved in water was estimated by UV absorption measurements, which allowed us to determine the adsorption capacity q_e_ expressed as milligrams of adsorbed phenol per total grams of GB sample (mg_phen_/g_GB_) (Figure 5b). In this regards, literature demonstrates the suitability of biopolymeric beads as effective adsorbents for phenolic compound removal.^[^ ^11^, ^81^ ^]^ Figure 5b summarizes the q_e_ results at variable times for geopolymerized beads. It should be noted that alginate/HNT beads before geopolymerization do not evidence absorption capacity towards phenol. In general, GP_GB_5s exhibited better removal efficiencies with respect to GP_GB_30 min. After 14.5 hours, we estimated that GP_GB_5s presents a q_e_ value of 0.58, which is ca. 1 order of magnitude larger compared to GP_GB_30 min (q_e_ = 0.079). The bead obtained by 5 second of NaOH treatment reached the maximum phenol absorption (q_e_ = ca. 1.5) within 25 hours. In contrast, the GP_GB_30 min sample reached its adsorption plateau (q_e_ = ca. 0.64) after 67 hours. These results are consistent with the absorption data toward dodecane vapours.

Regarding GP_GB_5s, we conducted a cyclic test after 25 hours of phenol removal from aqueous medium. The removal efficiency of the regenerated bead is 72.4% as compared with that determined in the first adsorption experiment. On this basis, we can conclude that the geopolymerized bead can be reused for adsorption of hydrocarbons although their removal performance is partly reduced after the regeneration process.

### 3.5 CO_2_ Capture Efficiency of Gel Beads

**Figure 6:**
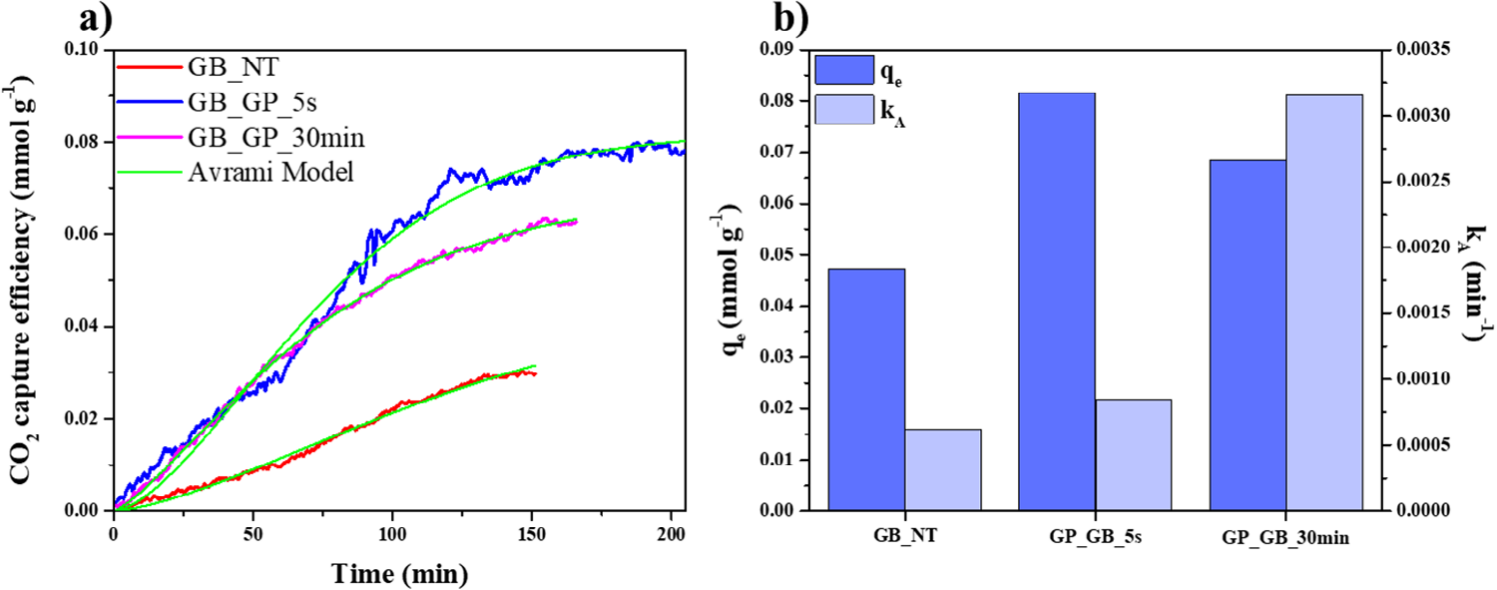
CO_2_ capture efficiency a) as a function of time for GB_NT, GP_GB_5s, and GP_GB_30 min samples. b) Adsorption parameters (q_e_, k_A_) obtained by the fitting analysis using Avrami model. The relative error is < 0.5%.

We investigated the effect of the geopolymerization on the CO_2_ capture capacity of alginate/HNTs beads by thermogravimetry. **Figure** **6**a shows the CO_2_ absorption efficiency as a function of time for GB_NT, GP_GB_5s, and GP_GB_30 min samples. The adsorption kinetic data were fitted using the Avrami Kinetics model (Equation (2)). Originally developed to describe nucleation and growth kinetics,^[^ ^82^ ^]^ this model has been effectively adapted to simulate CO_2_ adsorption behavior in various solid matrices. This includes applications to porous geopolymers,^[^ ^23^, ^24^ ^]^ lithium silicates sourced from demolition wastes,^[^ ^83^ ^]^ and metal‐organic frameworks.^[^ ^84^ ^]^ The Avrami Kinetics model is represented by the following equation

(3) $$\;q_{t}=q_{e}\;\;\left(1-e^{{\left(k_{A}t\right)}^{n_{A}}}\right)$$

where q_t_ is the adsorption value at time t, q_e_ is the adsorption value at equilibrium, k_A_ is the Avrami rate constant, and n_A_ is the Avrami parameter related to the induction time, where a higher parameter value indicates longer induction times.^[^ ^85^ ^]^

The obtained fitting parameters (q_e_ and k_A_) are collected in Figure 6b. The larger q_e_ values of GP_GB_5s and GP_GB_30 min samples distinctly indicated that the geopolymerization significantly improves the CO_2_ capture of alginate/HNT beads. This increased capacity can likely be attributed to the unique morphology and greater porosity characteristic of the geopolymerized samples. Notably, the q_e_ values for GP_GB_5s and GP_GB_30 min are 0.0817 mmol g^−1^ and 0.0 6843 mmol g^−1^, respectively, whereas the untreated GB_NT reaches a considerably lower value of 0.0 4725 mmol g^−1^. In terms of kinetic behaviour, the k_A_ parameter underscores that the geopolymerized samples exhibit faster CO_2_ uptake compared to the untreated sample, indicating a more rapid adsorption process. Particularly, GP_GB_30 min displays a much higher k_A_ value in respect of GP_GB_5s. The curves for all samples also reveal distinct induction periods, which are quantitatively confirmed by the Avrami exponent n_A_ values, recorded as 1.492 for GB_NT, 1.590 for GP_GB_5s, and 1.3106 for GP_GB_30 min. This range of n_A_ values suggests a combination of adsorption mechanisms, likely involving both chemisorption and physisorption processes, as noted in other studies.^[^ ^24^, ^86^ ^]^

## 4 Conclusion

We designed a novel procedure to obtain hollow spherical capsules (diameter of ca. 0.1 cm) by alkaline treatment of alginate beads loaded with halloysite clay nanotubes (HNTs). Firstly, we prepared composite beads with high halloysite content (50 wt%) by using the dipping method. Then, alginate/HNTs beads were immersed in NaOH solution (12 M) for different times (5 seconds and 30 minutes) to induce geopolymerization of halloysite clay. Both thermogravimetric analysis and FT‐IR spectroscopy revealed the alkaline treatment for a short time (5 seconds) is successful for the conversion of clay to geopolymeric material. Interestingly, the geopolymerization process altered the morphology of alginate/HNTs beads from a compact structure to a hollow spherical shape. According to the preparation protocol and SEM observations, the formation of the internal cavity within the bead is the combination of different mechanisms, which are related to variable crosslinking degree of alginate and inhomogeneous distribution of halloysite nanotubes that are concentrated in the bead core.

As expected by their hollow spherical morphology, the geopolymerized beads evidenced improved compressive properties (in terms of elasticity) and better water uptake capacity, which can be useful for moisture regulation.

Within environmental applications, we tested the suitability of hollow spherical capsules as adsorbents of hydrocarbons (dodecane and phenol) from gaseous and aqueous phases. We observed that the beads treated for 5 seconds present the highest adsorption capacity towards dodecane vapours. The dodecane uptake of the geopolymerized beads range between 26 and 38% on the dependence of the NaOH immersion time. These values are larger in comparison with that of untreated alginate/HNTs bead (21%). Similar observations were detected by studying the removal efficiency of phenol from aqueous solvent.

Furthermore, we investigated the effect of the geopolymerization on the CO_2_ capture capacity of the beads. According to previous results, the geopolymerized beads evidenced better performances in the CO_2_ sorption confirming that the hollow spherical capsules are efficient containers of gas molecules. As observed for dodecane adsorption, the highest CO_2_ capture amount (ca. 2 times larger than alginate/HNTs) was determined for short‐treated beads.

The versatility of the geopolymerized beads can be exploited within a wide range of environmental challenges, including air pollution mitigation, hydrocarbon spill management, and advanced water purification. In summary, geopolymerized gel beads offer a promising advancement in the field of adsorption materials. By tailoring the geopolymerization process, these materials can be optimized to address specific environmental needs effectively. These findings pave the way for further research and development, emphasizing their role in creating sustainable solutions to global environmental challenges.

## Conflict of Interest

The authors declare no conflicts of interest.

## Supporting information



Supporting Information

## Data Availability

The data that support the findings of this study are available from the corresponding author upon reasonable request.
