# Supplementary material for: Hollow Spherical Capsules From Geopolymerized Gel Beads With Halloysite Nanotubes for Pollutants Removal and CO2 Capture
Source: Small. 2025 Jun 17;21(33):2504306. doi: 10.1002/smll.202504306 (PMC12372448; doi:10.1002/smll.202504306)
Supplement: Supplementary file 1 — Supporting Information [file SMLL-21-2504306-s001.docx]

**Supporting Information**

**Hollow Spherical Capsules from Geopolymerized Gel Beads with Halloysite Nanotubes for Pollutants Removal and CO_2_ Capture**

Alessandro Lo Bianco^a^, Martina Maria Calvino^a^, Giuseppe Cavallaro*^,a^, Pavel Šiler^b^, Jaromìr Wasserbauer^b^, Stefana Milioto^a^, Giuseppe Lazzara^a^

Department of Physics and Chemistry “Emilio Segrè”, University of Palermo, Viale delle Scienze 17, 90128, Palermo, Italy. *[giuseppe.cavallaro@unipa.it](mailto:giuseppe.cavallaro@unipa.it)

Faculty of Chemistry, Institute of Materials Science, Brno University of Technology, Purkyňova 118, Brno 61200, Czech Republic


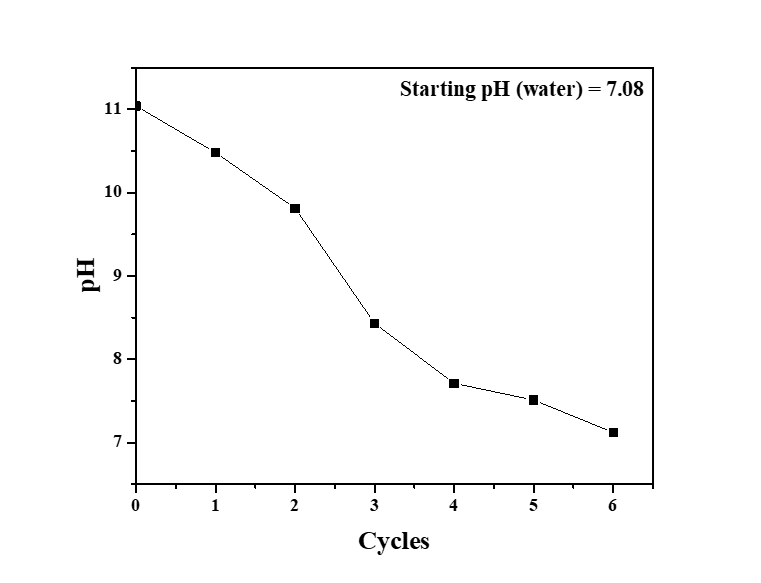


**Figure S1** pH of the rinsing water as a function of washing cycles for geopolymerized beads

**
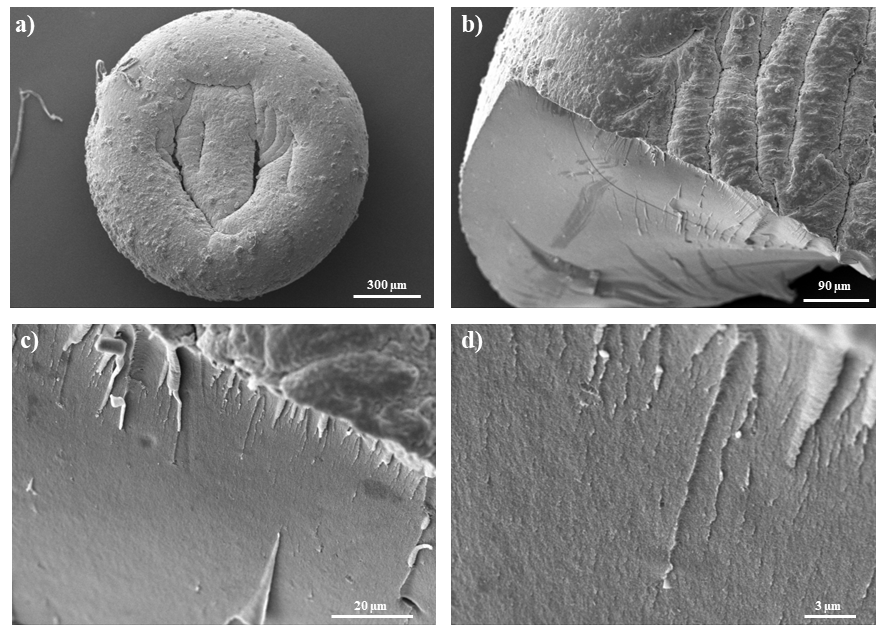
**

**Figure S2** SEM images of AB (a-b-c-d), for different parts of the sample, entire sample (a), cross-section (b) and matrix (c-d).


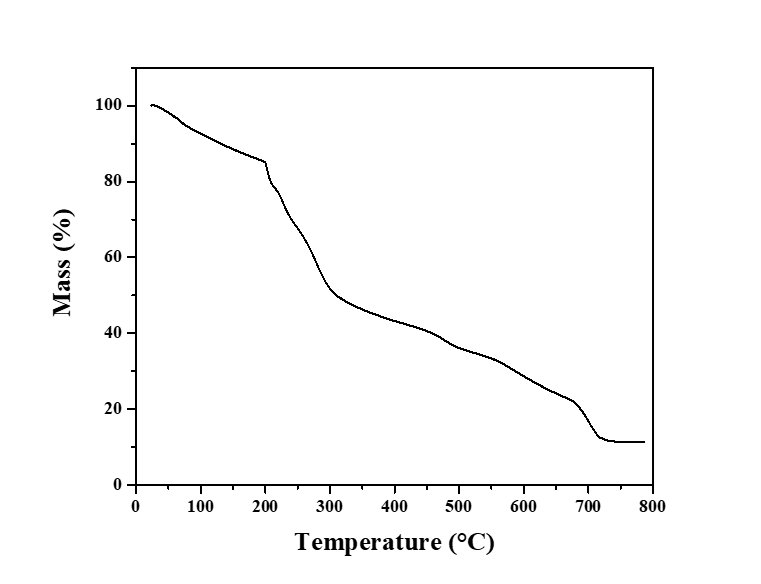


**Figure S3** Thermogravimetric curve of AB.

**Table S1.** Summary of IR absorption signals for the analyzed samples, including the corresponding wavenumber (cm⁻¹) and the associated vibrational modes.

| Signal | Wavenumber (cm^-1^) | Sample |
| --- | --- | --- |
| -OH vibrations | 3697, 3623 | GB_NT |
| -OH stretching | 3450 | All |
| -CH_3_ antisymmetric stretching | 2920 | All |
| C-H symmetric stretching | 2850 | All |
| H-O-H bending | 1635 | All |
| C=O stretching vibration | 1630 | All |
| Si-O, Si-O-Si stretching | 1090, 1044 | GB_NT |
| Si-O-T stretching | 1012 | GP_GB_5s, GP_GB_30min |
| Si-O-Si symmetric stretching | 805 | GB_NT |
| Si-O-Al stretching | 694, 753 | GB_NT |
| Si-O-Al bending | 720 | GP_GB_5s, GP_GB_30min |
| Al-O-Si deformation | 541 | GB_NT |
| Si-O-Si stretching | 465 | GP_GB_5s, GP_GB_30min |

**Table S2.** Energy Stored values obtained by integrating the static force vs strain curves.

| **Sample** | **Energy Stored (mJ/bead)** |
| --- | --- |
| AB | 0.59 ± 0.03 |
| GB_NT | 0.88 ± 0.04 |
| GP_GB_5s | 0.49 ± 0.02 |
| GB_GB_30min | 1.47 ± 0.07 |
